# Supplementary figures and images for: Blood flow guides sequential support of neutrophil arrest and diapedesis by PILR-β1 and PILR-α
Source: eLife. 2019 Aug 6;8:e47642. doi: 10.7554/eLife.47642 (PMC6699825; doi:10.7554/eLife.47642)

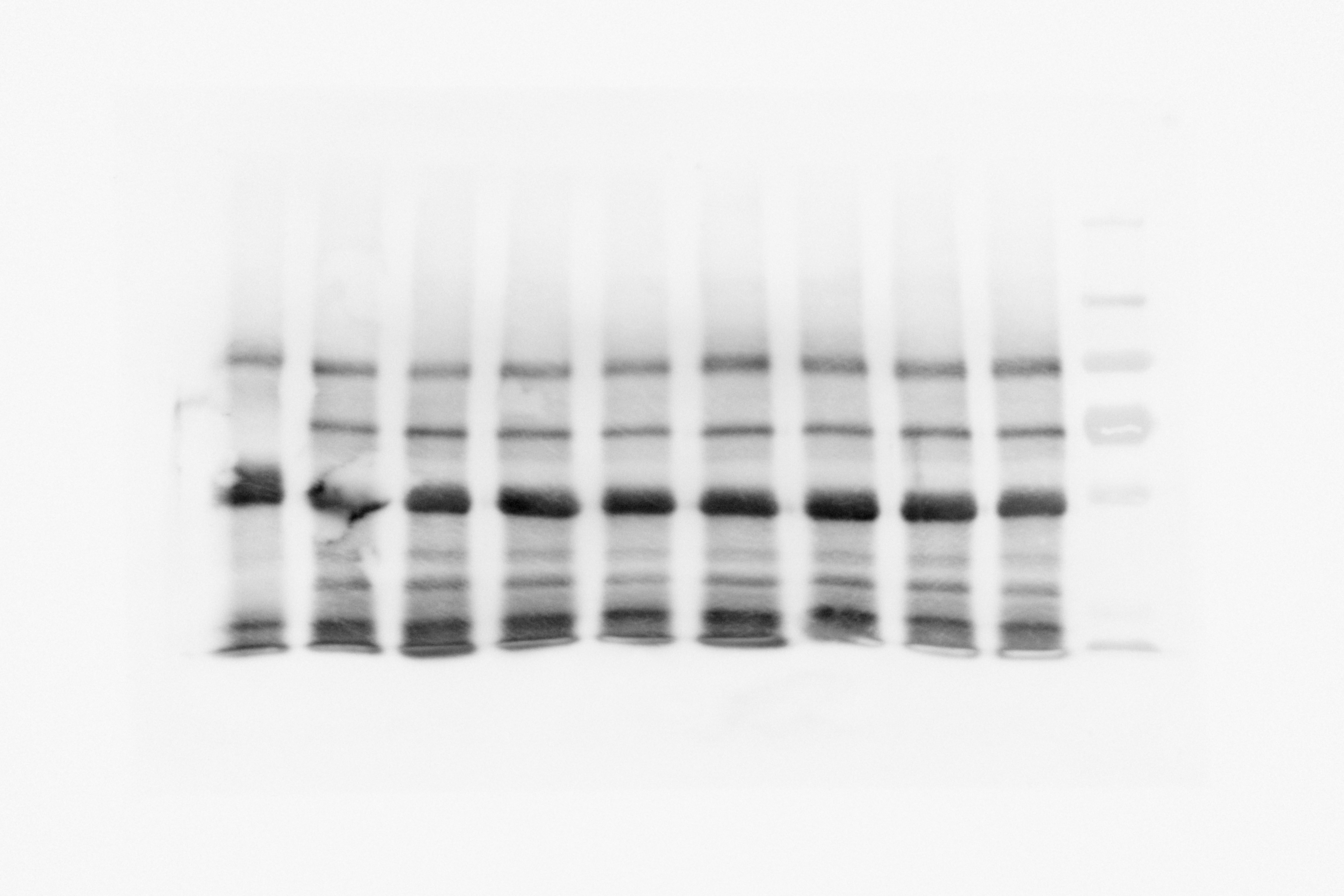

Supplement: Figure 3—figure supplement 1—source data 1. [file elife-47642-fig3-figsupp1-data1.zip › Figure 3-figure supplement 1-source data 1-2/Figure 3-figure supplement 1-source data 2 (Syk).tif]

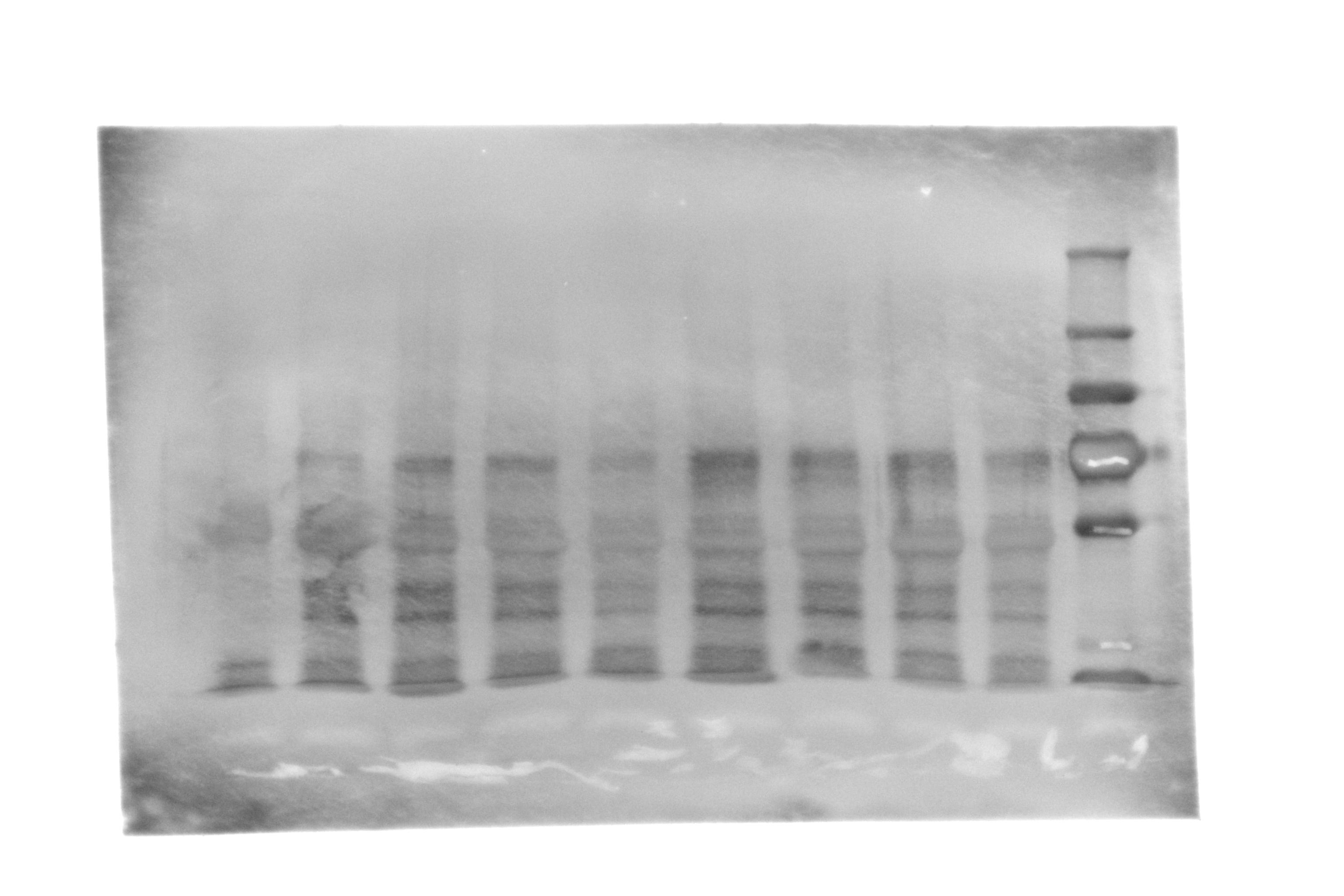

Supplement: Figure 3—figure supplement 1—source data 1. [file elife-47642-fig3-figsupp1-data1.zip › Figure 3-figure supplement 1-source data 1-2/Figure 3-figure supplement 1-source data 1 (4G10).tif]

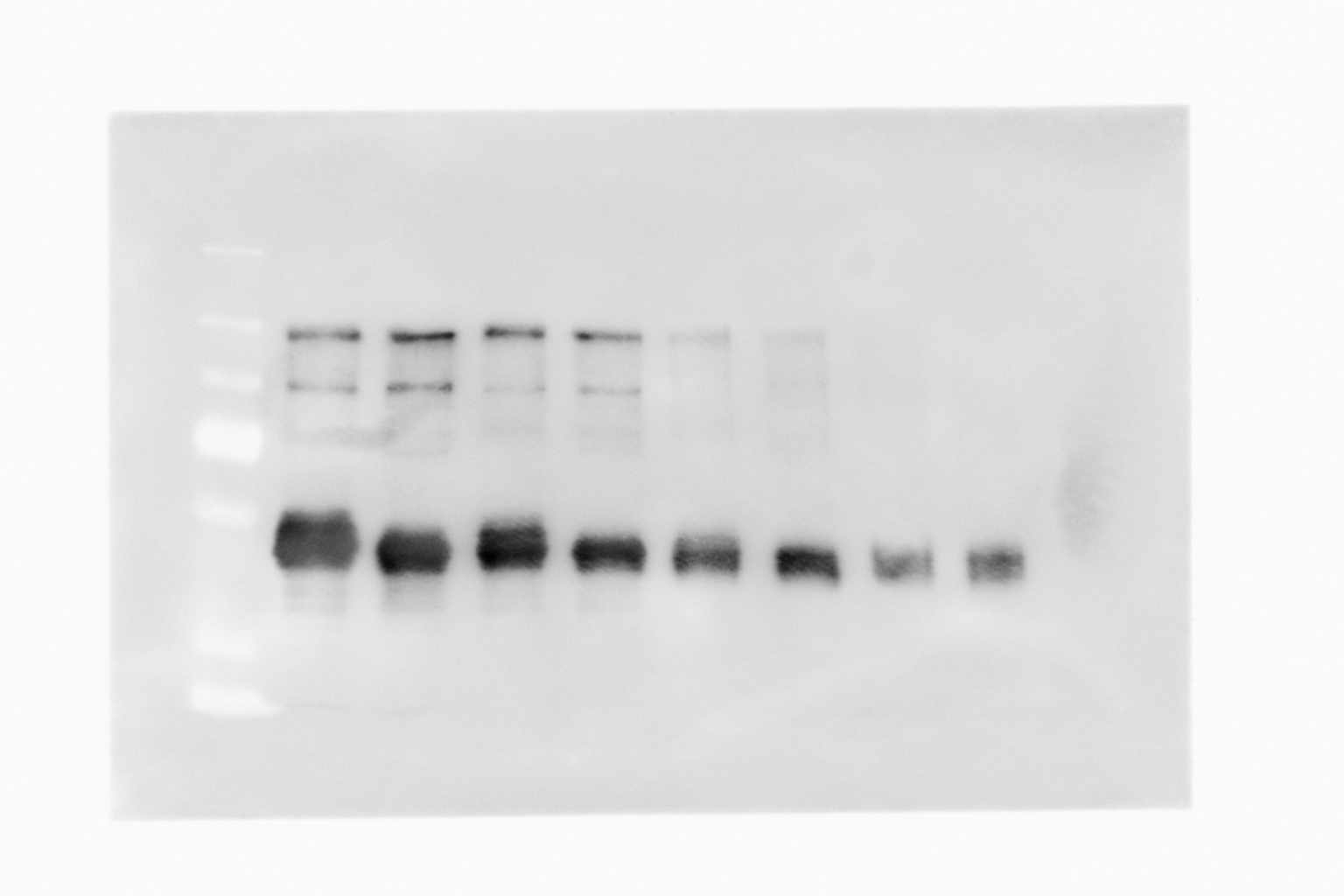

Supplement: Figure 5—figure supplement 2—source data 1. [file elife-47642-fig5-figsupp2-data1.zip › Figure 5-figure supplement 2-source data 1-2/Figure 5-figure supplement 2-source data 1 (A).tif]

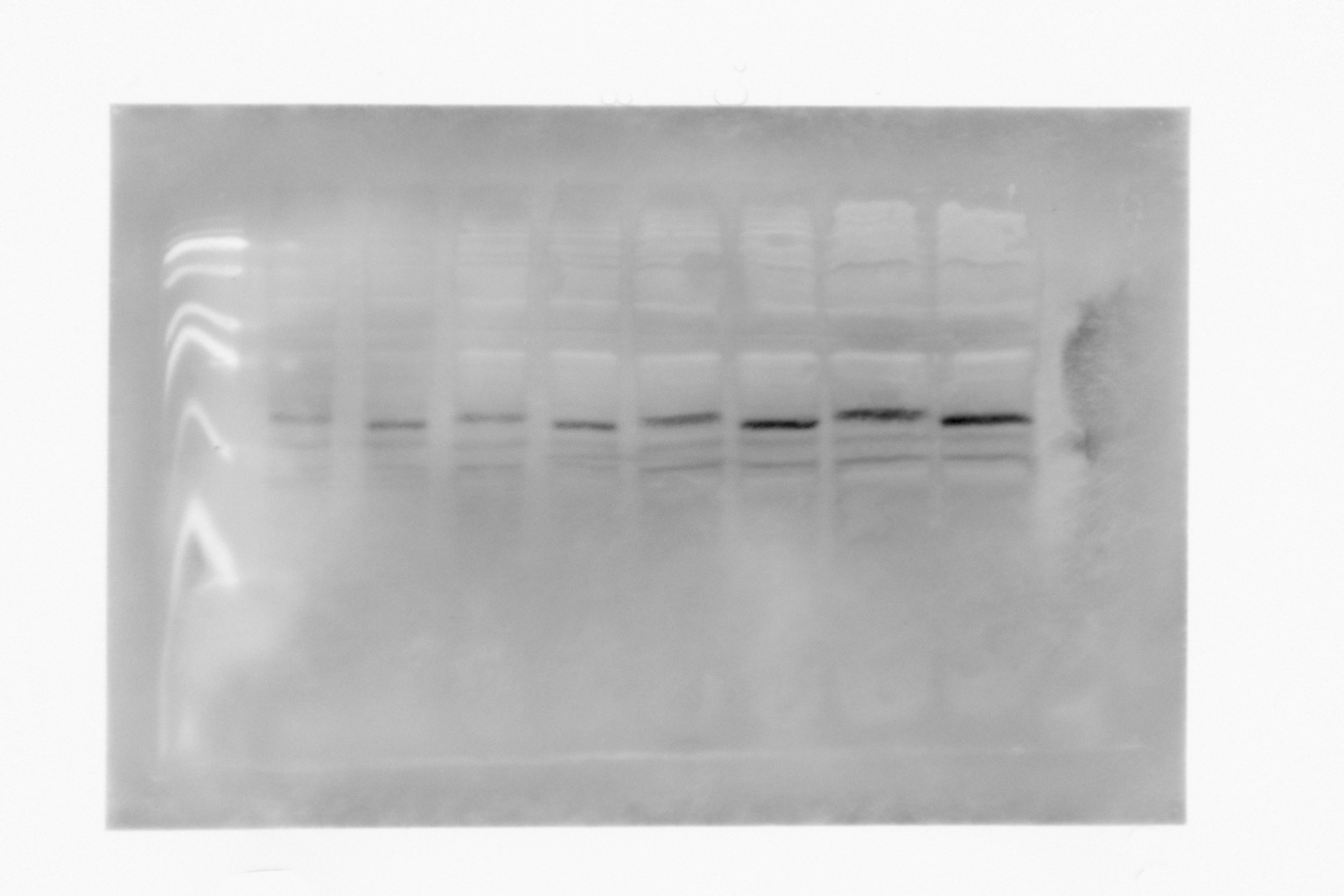

Supplement: Figure 5—figure supplement 2—source data 1. [file elife-47642-fig5-figsupp2-data1.zip › Figure 5-figure supplement 2-source data 1-2/Figure 5-figure supplement 2-source data 2 (B).tif]
